# Adipose-derived stem cell-conditioned medium mitigates ischemia-induced neuronal injury via the JAK1/STAT3 signaling pathway

**DOI:** 10.3389/fncel.2026.1744887

**Published:** 2026-02-23

**Authors:** Huan Huang, Mouwei Zheng, Yongxing Lai, Yixian Zhang, Yan Chen, Nan Liu

**Affiliations:** 1 Department of Neurology, Fujian Medical University Union Hospital, Fuzhou, Fujian, China; 2 Department of Geriatric Medicine, Fuzhou University Affiliated Provincial Hospital, Fuzhou, Fujian, China; 3 Fujian Provincial Institute of Clinical Geriatrics, Fuzhou, Fujian, China; 4 Department of Rehabilitation, Fujian Medical University Union Hospital, Fuzhou, Fujian, China

**Keywords:** adipose-derived stem cells, conditioned medium, ischemic stroke, JAK1/STAT3 signaling, mitochondrial function, neurovascular regeneration, paracrine effect, synaptic plasticity

## Abstract

**Introduction:**

Adipose-derived stem cells (ADSCs) demonstrate therapeutic potential for ischemic stroke, primarily through paracrine actions. However, the specific intracellular signaling pathways underlying these benefits remain unclear. This study investigates the critical role of JAK1/STAT3 signaling in neuroprotection mediated by ADSC-conditioned medium (ADSC-CM).

**Methods:**

We employed a dual-model approach. *In vivo,* rats subjected to transient middle cerebral artery occlusion (tMCAO) received intravenous ADSC-CM or vehicle at 2, 24, and 48 h post-ischemia, with or without the JAK1 inhibitor GLPG0634. Neurological function was evaluated over a period of 7 days. Subsequently, infarct volume, brain edema, neuronal survival, neurovascular regeneration, synaptic ultrastructure, mitochondrial function, and energy metabolism were analyzed. *In vitro*, primary cortical neurons subjected to oxygen–glucose deprivation (OGD) were treated with ADSC-CM with or without GLPG0634 to assess neurite outgrowth. Activation of the JAK1/STAT3 pathway was confirmed by Western blot in both models.

**Results:**

*In vivo*, ADSC-CM significantly improved neurological function, reduced infarct volume and brain edema, and enhanced neuronal survival, nerve fiber regeneration, angiogenesis, synaptic plasticity, and mitochondrial function in tMCAO rats. *In vitro*, ADSC-CM promoted neurite outgrowth in OGD-injured neurons. Crucially, all these multifaceted neuroprotective effects were completely abolished by co-treatment with GLPG0634. Mechanistically, ADSC-CM robustly activated JAK1 and STAT3 phosphorylation in both models, an effect effectively inhibited by GLPG0634.

**Discussion:**

The neuroprotective effects of ADSC-CM are mechanistically linked to the activation of the JAK1/STAT3 pathway, which mitigates ischemic damage by promoting neuronal salvage, neurovascular regeneration, synaptic plasticity, and metabolic recovery, thereby enhancing neurological functional recovery after stroke.

## 1 Introduction

Ischemic stroke, characterized by acute cerebral blood flow disruption and subsequent neuronal death, stands as a leading cause of global mortality and long-term disability (He et al., 2024; Ya et al., 2024). Current reperfusion strategies, including thrombolysis and mechanical thrombectomy, are constrained by strict eligibility criteria, particularly the narrow therapeutic time window (Chen et al., 2023; Zhu et al., 2025). Furthermore, they fail to address secondary injury cascades triggered by reperfusion, which induce multiple pathophysiological processes, including intracellular calcium overload, oxidative stress, free radical formation, cellular acidosis, and neuroinflammatory processes (Kim et al., 2025; Puzio et al., 2023; Parvardeh et al., 2022). These secondary injury cascades exacerbate the initial ischemic damage, leading to further neuronal injury and hindering functional recovery. This highlights the limitations of current therapies and underscores the pressing need for novel approaches that target both acute neuroprotection and subsequent neural repair mechanisms, which could potentially improve functional recovery and reduce disability following ischemic stroke (Guo J. et al., 2024).

Adipose-derived stem cells (ADSCs), a subpopulation of mesenchymal stem cells (MSCs), have emerged as a promising cell-based therapy for ischemic stroke due to their multifaceted regenerative capacities (Zhou B. et al., 2024; Song et al., 2025). ADSCs have unique advantages over MSCs from other sources. They are derived from abundant adipose tissue, which can be easily harvested via minimally invasive liposuction without ethical concerns. Moreover, their vigorous paracrine activity and low immunogenicity support stable proliferation and differentiation with few adverse effects (Zhou B. et al., 2024; Zhang Y. C. et al., 2022). The therapeutic efficacy of ADSCs is mainly attributed to their paracrine effect, which can upregulate a lot of key mediators, including neurotrophic factors (e.g., brain-derived neurotrophic factor [BDNF] and nerve growth factor [NGF]), anti-apoptotic proteins (e.g., Bcl-2 and X-linked inhibitor of apoptosis protein [XIAP]), anti-inflammatory cytokines (e.g., interleukin-10 [IL-10] and transforming growth factor-β1 [TGF-β1]), and pro-angiogenic molecules (e.g., vascular endothelial growth factor [VEGF], angiogenin-1 [Ang-1]) (Zangiabadi et al., 2024; Baldassarro et al., 2023; Seo et al., 2017; Lu et al., 2011; Phelps et al., 2023; Giannasi et al., 2024). This coordinated upregulation drives multimodal recovery mechanisms, including reduced neuronal apoptosis, enhanced neurovascular remodeling, and improved functional outcomes in experimental stroke models (Zhang Y. C. et al., 2022). Furthermore, these paracrine factors demonstrate clinical translation potential due to their ability to cross the blood–brain barrier (BBB), avoid first-pass metabolism, reduce the risk of microvascular thrombosis, and enable large-scale production (Silvana et al., 2024). However, the intracellular signaling mechanisms linking ADSC-derived paracrine factors to structural and functional restoration remain poorly defined, posing a major barrier to clinical translation.

The Janus kinase/signal transducer and activator of transcription (JAK/STAT) signaling pathway, an essential regulator of cytokine and growth factor signaling, mediates a range of physiological and pathological processes, including cellular proliferation, differentiation, survival dynamics, inflammatory responses, and immune regulation (Dodington et al., 2018; Xin et al., 2020). The JAK/STAT pathway exhibits context-dependent duality in its role within the pathogenesis of ischemic stroke. STAT3, a critical downstream mediator, exhibits paradoxical neuroprotective and neurotoxic effects contingent upon specific cellular microenvironmental cues. Experimental evidence confirms that STAT3 activation exerts pro-survival effects through the suppression of neuronal apoptosis (Zuo et al., 2023; Niu et al., 2023), mitigation of oxidative damage (Xi et al., 2025), and stimulation of angiogenesis (Jia et al., 2022), collectively contributing to reduced infarct volume and neurological recovery (Lin et al., 2024). Conversely, other studies demonstrate that STAT3 activation may exacerbate neuroinflammation and neuronal death under certain conditions (Zhang S. S. et al., 2022; Zhang et al., 2024). This functional dichotomy highlights the need to identify STAT3’s upstream modulators and context-specific downstream effectors. Although STAT3 activation mediates neural repair processes triggered by BDNF- or IL-10-based therapies (Lin et al., 2006; Chen et al., 2016), its mechanistic involvement in ADSC-mediated neuroprotection remains an uncharted territory that requires systematic investigation.

Existing research on ADSCs therapy for stroke primarily focuses on their differentiation potential and broad functional outcomes, with limited exploration of pathway-specific mechanisms (Ya et al., 2024; Zhou B. et al., 2024). Additionally, conflicting reports regarding STAT3’s role in stroke emphasize the necessity to delineate its regulatory mechanisms in the context of ADSC-based interventions. Specifically, it remains unclear whether ADSC-conditioned medium (ADSC-CM) coordinates structural and functional recovery after ischemic stroke through the JAK1/STAT3 signaling pathway. To address these gaps, our study employs a dual-model approach that combines *in vitro* oxygen–glucose deprivation (OGD) of primary cortical neurons with *in vivo* transient middle cerebral artery occlusion (tMCAO) in rats. We systematically evaluate the effects of ADSC-CM on infarct volume, brain edema, neuronal survival, nerve fiber and blood vessel regeneration, synaptic and mitochondrial ultrastructure, energy metabolism, and neurological functional recovery. Pharmacological inhibition of JAK1 with GLPG0634 establishes a causal link between JAK1/STAT3 activation and the observed neuroprotection.

By integrating these experimental paradigms, our work seeks to elucidate the mechanism of ADSC-CM-mediated neuroprotection and demonstrate the role of JAK1/STAT3 as a critical signaling axis. These findings not only enhance the translational potential of ADSCs by providing a molecular rationale for their therapeutic effects but also pave the way for the development of combinatorial strategies to optimize JAK1/STAT3 modulation in stroke rehabilitation.

## 2 Materials and methods

### 2.1 Isolation, culture, and identification of ADSCs

ADSCs were isolated from the subcutaneous adipose tissue of four-week-old male Sprague–Dawley (SD) rats (SPF grade, weighing 80–100 g), as this age yields cells with high proliferative capacity and stable stemness (Yang et al., 2022; Guo W. et al., 2024). The isolation procedure was carried out according to a previous method (Yang et al., 2022), with minor modifications. The adipose tissue was first washed with betadine and then finely minced (< 1 mm^3^) in phosphate-buffered saline (PBS). The minced tissue was digested with 0.3% type I collagenase (Sigma-Aldrich, St. Louis, MO, USA) at 37 °C for 1 h under gentle agitation. After digestion, the tissue was filtered through a 100-mesh sieve and centrifuged at 1000 rpm for 10 min to obtain the cell pellet. The pellet was resuspended in growth medium composed of Dulbecco’s Modified Eagle’s Medium (DMEM; Gibco, Grand Island, NY, USA) supplemented with 100 U/mL penicillin (Gibco), 100 μg/mL streptomycin (Gibco), and 10% fetal bovine serum (FBS; Gibco), and then incubated at 37 °C in a humidified atmosphere of 5% CO₂ and 95% air for 24 h. Non-adherent cells and debris were removed after the initial incubation period, and the adherent cells were maintained in fresh growth medium. When the cells reached 80% confluence, they were passaged using 0.05% trypsin (Sigma-Aldrich). Cells at passages 3–4 were used for subsequent experiments. The identity of the ADSCs was confirmed by their characteristic morphology and expression of specific surface markers. For immunofluorescence staining, cells were seeded onto 24-well plates and fixed with 4% paraformaldehyde. After washing with PBS, the cells were blocked with 5% normal goat serum (Beyotime, Jiangsu, China) for 30 min, followed by incubation with primary mouse antibodies against CD34 (1:200, sc-65261, Santa Cruz Biotechnology, Santa Cruz, CA, USA), CD44 (1:200, sc-53068, Santa Cruz Biotechnology), CD45 (1:200, sc-1178, Santa Cruz Biotechnology), and CD105 (1:200, sc-20072, Santa Cruz Biotechnology) for 1 h. The cells were then rewashed and incubated with Cy3-conjugated goat anti-mouse IgG (1:400, AS008, ABclonal, Wuhan, China) for 45 min. Finally, the cells were visualized under a fluorescence microscope (Eclipse 80i, Nikon, Tokyo, Japan).

### 2.2 Preparation of conditioned medium (CM) derived from ADSCs

To avoid potential confounding effects of the JAK1 inhibitor GLPG0634 on the viability and proliferation of ADSCs, CM derived from ADSCs was used to investigate ADSC-mediated neuroprotective molecular pathways. Briefly, ADSCs were washed twice with serum-free DMEM/F12 (Gibco), and the culture medium was replaced with fresh serum-free DMEM/F12. After 48 h of incubation, the CM was collected and centrifuged at 4 °C and 1,500 rpm for 10 min to remove cell debris. This was followed by a 40-fold concentration using a centrifugal filter device (Millipore, Bedford, MA, USA) through additional centrifugation at 4 °C and 6,000 rpm for 30 min. As a vehicle control, serum-free DMEM/F12 was subjected to the same 40-fold concentration process to ensure equivalent experimental conditions. Both concentrated ADSC-CM and serum-free DMEM/F12 were stored at −80 °C until use.

### 2.3 Establishment of the tMCAO model

Transient middle cerebral artery occlusion (tMCAO) was induced by creating an intraluminal vascular occlusion, as previously reported (Zhang et al., 2021). Eight-week-old male SD rats (SPF grade), weighing 250–280 g, were anesthetized with isoflurane (4% for induction, 2% for maintenance in 30% O₂ and 70% N₂O). The right common carotid artery (CCA), external carotid artery (ECA), and internal carotid artery (ICA) were exposed via a midline neck incision. The CCA was temporarily occluded with a vascular clamp, and the ECA was ligated and severed. A silicon-coated 3–0 monofilament nylon suture (diameter: 0.37 ± 0.02 mm) was inserted through the severed ECA into the ICA and advanced 18–20 mm until it reached the origin of the middle cerebral artery (MCA), effectively blocking blood flow. The filament was left in place for 1.5 h to induce ischemia. After the occlusion period, the filament was slowly withdrawn, the ECA was ligated, the vascular clamp was removed from the CCA, and reperfusion of the MCA was restored. The neck incision was then closed with sutures. Throughout the procedure, the rats’ body temperature was maintained using a heating lamp and a heating pad. Inclusion in the study was determined using Longa’s five-point scale (Longa et al., 1989), with only rats scoring 1 to 3 being enrolled. Exclusion criteria included inactivity, subarachnoid hemorrhage, signs of severe dehydration, abnormal mucous membrane color, abnormal respiratory patterns, or death during the experimental procedure. Any excluded animal was replaced to ensure the target sample size was maintained.

### 2.4 Intravenous administration of ADSC-CM combined with pharmacological blockade of JAK1/STAT3 pathway in tMCAO-treated rats

Rats were infused with 100 μL of 40-fold concentrated ADSC-CM or serum-free DMEM/F12 (as a vehicle) via the tail vein at 2, 24, and 48 h post-tMCAO, for three consecutive days. To pharmacologically inhibit JAK1 phosphorylation, GLPG0634 (Selleck Chemicals, Houston, TX, USA) was dissolved in ADSC-CM or vehicle to a final concentration of 20 nM. Rats were randomly assigned to four groups: (1) tMCAO+vehicle, (2) tMCAO+CM (ADSC-CM), (3) tMCAO+CM + GLPG (ADSC-CM + GLPG0634), and (4) tMCAO+GLPG (vehicle + GLPG0634). Neurological function was assessed at baseline and 1, 3, and 7 days post-tMCAO. Upon completion of behavioral testing, rats were deeply anesthetized by isoflurane overdose, followed by transcardial perfusion, and brain tissues were extracted for histological and molecular analyses. The peri-infarct cortex (ischemic penumbra) was selected as the region of interest for all analyses because it represents the primary target for neuroprotective interventions and the key site where treatments such as ADSC-CM are expected to exert their salvaging and restorative effects (Alasoadura et al., 2024; Hazime et al., 2021).

### 2.5 Isolation, culture, and identification of primary cortical neurons

Primary cortical neurons were isolated from embryonic day 16–18 SD rat embryos following a previously established protocol, with minor modifications (Chen et al., 2016). The cerebral cortex of embryos was dissected, finely minced into approximately 1 mm^3^ pieces, and transferred to tubes containing 0.25% trypsin–EDTA (Gibco). The tissue was incubated at 37 °C for 15 min, with gentle agitation every 5 min. The trypsinization was terminated by adding 5 mL of 20% FBS, after which the tissue was gently triturated using a Pasteur pipette. The cell clumps were allowed to settle for 2 min to permit debris to precipitate, and the supernatant was collected and filtered through a 75 μm pore-sized filter. The filtrate was centrifuged at 1000 rpm for 2 min. The cell pellet was resuspended in Neurobasal medium (Gibco) supplemented with 2% B27 (Gibco), 0.5 mM glutamine (Gibco), and 50 U/mL penicillin/streptomycin (Gibco). For immunofluorescence and Western blot analyses, 3 × 10^5^ and 3 × 10^6^ cells, respectively, were plated on poly-L-lysine-coated (0.05 mg/mL) 24 mm × 24 mm coverslips and cultured at 37 °C with 5% CO_2_. After 24 h, the initial medium was replaced, and thereafter, half of the medium was refreshed every 3 days. Neuronal identity and purity were confirmed by immunofluorescence staining with an antibody against the neuronal-specific marker class III β-tubulin (Tuj1).

### 2.6 Establishment of the OGD model

The OGD model was established as previously described with slight modifications (Thiebaut et al., 2021). On the fifth day *in vitro*, primary cortical neurons were washed with glucose-free DMEM (Gibco) and cultured in the same medium. The cells were then incubated in an anaerobic chamber containing 5% CO_2_ and 95% N_2_ at 37 °C for 90 min. After the OGD period, the glucose-free DMEM was replaced with the original culture medium, and the cells were returned to a normoxic chamber containing 5% CO_2_ at 37 °C for reperfusion periods.

### 2.7 ADSC-CM treatment combined with pharmacological blockade of the JAK1/STAT3 pathway in OGD-injured neurons

After OGD injury, cortical neurons were treated with 50 μL of 40-fold concentrated ADSC-CM or serum-free DMEM/F12 (as a vehicle). To pharmacologically inhibit JAK1 phosphorylation, neuronal cultures were treated with GLPG0634 at a final concentration of 20 nM. The experimental groups were as follows: (1) OGD + vehicle, (2) OGD + CM (ADSC-CM), (3) OGD + CM + GLPG (ADSC-CM + GLPG0634), and (4) OGD + GLPG (vehicle + GLPG0634). At 12 h post-treatment, neuronal expression levels of JAK1, phosphorylated-JAK1 (pJAK1), STAT3, and phosphorylated-STAT3 (pSTAT3) were analyzed by western blot. At 48 h post-treatment, neuronal morphometric parameters, including the length of the longest neurite and the number of primary neurites, were quantified using Tuj-1 immunofluorescence staining.

### 2.8 Neurobehavioral tests

All rats were able to perform the neurobehavioral tests and did not exhibit significant asymmetries before tMCAO. Neurobehavioral tests were conducted by an investigator blinded to the treatment groups using the modified neurological severity scores (mNSS) method (Chen et al., 2019). The mNSS scoring system evaluates motor function (muscle status and abnormal movement), sensory response (visual, tactile, and proprioceptive), reflex behavior (pinna, corneal, and startle reflexes), and balance. In this system, a score of 0 indicates no neurological deficit, while higher scores reflect increasing severity of neurological impairment, with a maximum possible score of 18. Each failed test or absent reflex counts as 1 point toward the total score.

### 2.9 Western blot

Proteins were extracted from peri-infarct cortex tissues of rats and primary cortical neurons using RIPA lysis buffer (Beyotime) according to the manufacturer’s instructions. Protein concentrations were determined using the BCA Protein Assay Kit (Beyotime). Equal amounts of protein (20 μg per sample) were loaded onto a 10% SDS-PAGE gel for electrophoresis and subsequently transferred to polyvinylidene fluoride (PVDF) membranes (Millipore). The membranes were blocked with 5% bovine serum albumin (BSA) in tris-buffered saline containing 0.1% Tween 20 (TBS-T) at room temperature for 2 h, followed by overnight incubation at 4 °C with primary antibodies: mouse anti-JAK1 antibody (1:1000, 66466-1-Ig, Proteintech, Chicago, IL, USA), rabbit anti-pJAK1 antibody (1:1000, AF2012, Affinity, Jiangsu, China), rabbit anti-STAT3 antibody (1:1000, 10253-2-AP, Proteintech), rabbit anti-pSTAT3 antibody (1:1000, AF3293, Affinity), and mouse anti-GAPDH antibody (1:1000, AF0006, Beyotime). After washing, membranes were incubated with secondary antibodies at room temperature for 2 h: horseradish peroxidase-conjugated goat anti-mouse IgG (1:2000, GB23301, Servicebio, Wuhan, China) and horseradish peroxidase-conjugated goat anti-rabbit IgG (1:2000, GB23303, Servicebio). Signals were detected using enhanced chemiluminescence (Beyotime) and quantified by ImageJ software (National Institutes of Health, Bethesda, MD, USA). Protein expression data were normalized to GAPDH as a loading control, with one sample from either the tMCAO+vehicle or OGD + vehicle group serving as the calibrator (1 × expression).

### 2.10 Nissl staining analysis

To assess infarct volume, brain edema, and neuronal survival, coronal brain sections were prepared and stained with Nissl stain. Briefly, sections were washed three times in PBS for 5 min each, then incubated in 0.1% cresyl violet acetate (Servicebio) for 10 min at room temperature. After staining, sections were rinsed in distilled water and differentiated in 70% ethanol for 5–10 s under microscopic observation until Nissl bodies were clearly visible. Following this, sections were dehydrated through graded ethanol (70, 95, and 100%) for 2 min each, and then cleared twice in fresh xylene for 5 min each. Finally, sections were mounted in balsam and imaged under a light microscope. Neuronal survival was evaluated based on established cytological criteria (Sun et al., 2009): Survival neurons exhibited a distinct nucleus and nucleolus, along with well-preserved Nissl substance in the cytoplasm, whereas necrotic neurons were identified by the loss of Nissl bodies, chromatolysis, nuclear pyknosis, eosinophilic cytoplasm, or the absence of cellular structure, often accompanied by reduced intercellular space and intense staining. Quantitative analysis was performed on five equidistant sections (collected every 2 mm starting 3–4 mm from the frontal pole) using ImageJ software. Infarct volume was calculated as the ratio of infarct area to total brain area, averaged across sections. Brain edema was quantified as the relative volume difference between hemispheres using the Kaplan method (Kuts et al., 2019): edema ratio = [(volume of ipsilateral hemisphere—volume of contralateral hemisphere) / volume of contralateral hemisphere] × 100%. Neuronal survival was quantified by analyzing two randomly selected, non-overlapping fields per section within the defined peri-infarct cortex, across five equidistant sections. The survival rate per field was calculated as the average ratio of Nissl^+^ neurons to total neurons. A single representative value per animal was then derived by averaging results across all fields and sections, ensuring a comprehensive assessment of the entire peri-infarct region.

### 2.11 Immunofluorescence staining analysis

Coronal sections (10 μm) from SD rats were fixed with 4% paraformaldehyde, permeabilized with 0.1% Triton X-100, and blocked with 5% normal goat serum. Sections were incubated with the following primary antibodies at 4 °C overnight: rabbit anti-neurofilament-200 (NF-200) antibody (1:100, A05307, Boster) for nerve fibers, and rabbit anti-CD31 antibody (1:100, GB11063-2, Servicebio) for blood vessels. After washing, sections were incubated with Cy3-conjugated goat anti-rabbit IgG (1:400) for 1 h in the dark. Every tenth section between Bregma +1.60 mm and −2.12 mm was analyzed (3 sections/brain). NF-200^+^ nerve fiber density (expressed as a percentage relative to one sample from the tMCAO+vehicle group) and CD31^+^ vessel density (counts/mm^2^) in peri-infarct cortex were measured from three randomly selected regions per section using ImageJ software. Final brain-level values were obtained by averaging results from three sections per brain.

Primary cortical neurons cultured on sterile coverslips were fixed with 4% paraformaldehyde for 15 min at room temperature, followed by three washes in PBS. Cells were blocked with 5% normal goat serum and permeabilized with 0.1% Triton X-100 (Sigma). After overnight incubation with rabbit anti-Tuj1 antibody (1:200, BM3881, Boster, Wuhan, China) at 4 °C, cells were washed and then incubated with Cy3-conjugated goat anti-rabbit IgG (1:400, AS007, ABclonal) for 1 h at room temperature in the dark. Nuclei were counterstained with 4,6-diamidino-2-phenylindole (DAPI) (1:1000, Sigma) for 10 min. Coverslips were mounted and imaged using fluorescence microscopy. The longest neurite length and primary neurite number were quantified using ImageJ software, with averages calculated from 10 randomly selected neurons across 4–5 fields per coverslip.

### 2.12 Transmission electron microscopy (TEM) analysis

Rat brains were carefully removed, and small tissue blocks of approximately 1 mm^3^ were dissected from the peri-infarct cortical region. The tissue blocks were fixed in 2.5% glutaraldehyde overnight at 4 °C, followed by three washes with PBS. Subsequently, the tissue was post-fixed in 1% osmium tetroxide for 2 h and washed three times with PBS. The tissue blocks were then dehydrated in a graded series of ethanol and acetone solutions. After dehydration, the tissue blocks were embedded in epoxy resin and polymerized at 60 °C for 48 h. The embedded blocks were trimmed, and ultrathin sections (70–90 nm thick) were cut using an ultramicrotome (UC7, Leica Microsystems GmbH, Wetzlar, Germany). The sections were stained with 3% uranyl acetate for 15 min and lead citrate for 10 min. Finally, the stained sections were examined and imaged using a TEM (JEM-1400, JEOL Ltd., Tokyo, Japan). The ultrastructural characteristics of synapses, including the number of synapses, thickness of postsynaptic densities (PSDs), length of synaptic active zones, and width of synaptic clefts, were quantified using ImageJ software. The integrity and morphology of mitochondria, such as swelling, membrane integrity, and cristae density, were also evaluated. All image analyses, including those mentioned above, were conducted by an independent investigator blinded to the treatment groups.

### 2.13 Energy metabolism assessment

Peri-infarct cortical tissue samples were excised, weighed, and transferred to a glass homogenizer. For each gram of tissue, 9 mL of ice-cold normal saline or double-distilled water was added (weight-to-volume ratio of 1:9). Homogenization was performed at 4 °C. The resulting homogenate was centrifuged at 3500 rpm for 10 min at 4 °C, and the supernatant was collected. Levels of ATP, Na^+^-K^+^-ATPase, and Ca^2+^-Mg^2+^-ATPase were measured using commercially available assay kits (Jiancheng Bioengineering Institute, Nanjing, China) according to the manufacturer’s instructions.

### 2.14 Statistical analysis

Statistical analyses were performed using SPSS 27.0 software (IBM Corporation, Armonk, NY, USA). Continuous data are expressed as mean ± SEM. The normality of data distribution was assessed using the Shapiro–Wilk test, and the homogeneity of variances across groups was confirmed using Levene’s test. Comparisons between multiple groups were analyzed using one-way or two-way analysis of variance (ANOVA), followed by Bonferroni’s *post hoc* test for multiple comparisons. Statistical significance was set at *p* < 0.05 (two-tailed). A post hoc power analysis was conducted for all outcome measures using G*Power 3.1.9.7 software (Heinrich Heine University Düsseldorf, Düsseldorf, Germany). This analysis was based on the observed effect sizes and variances derived from the experimental data, with a significance level (*α*) set at 0.05. The results demonstrated that the statistical power (1 - *β*) for the reported significant findings exceeded 90%, indicating that the sample size employed was adequate to detect the treatment effects with a high degree of reliability.

## 3 Results

### 3.1 Characteristics of ADSCs

**Figure 1:**
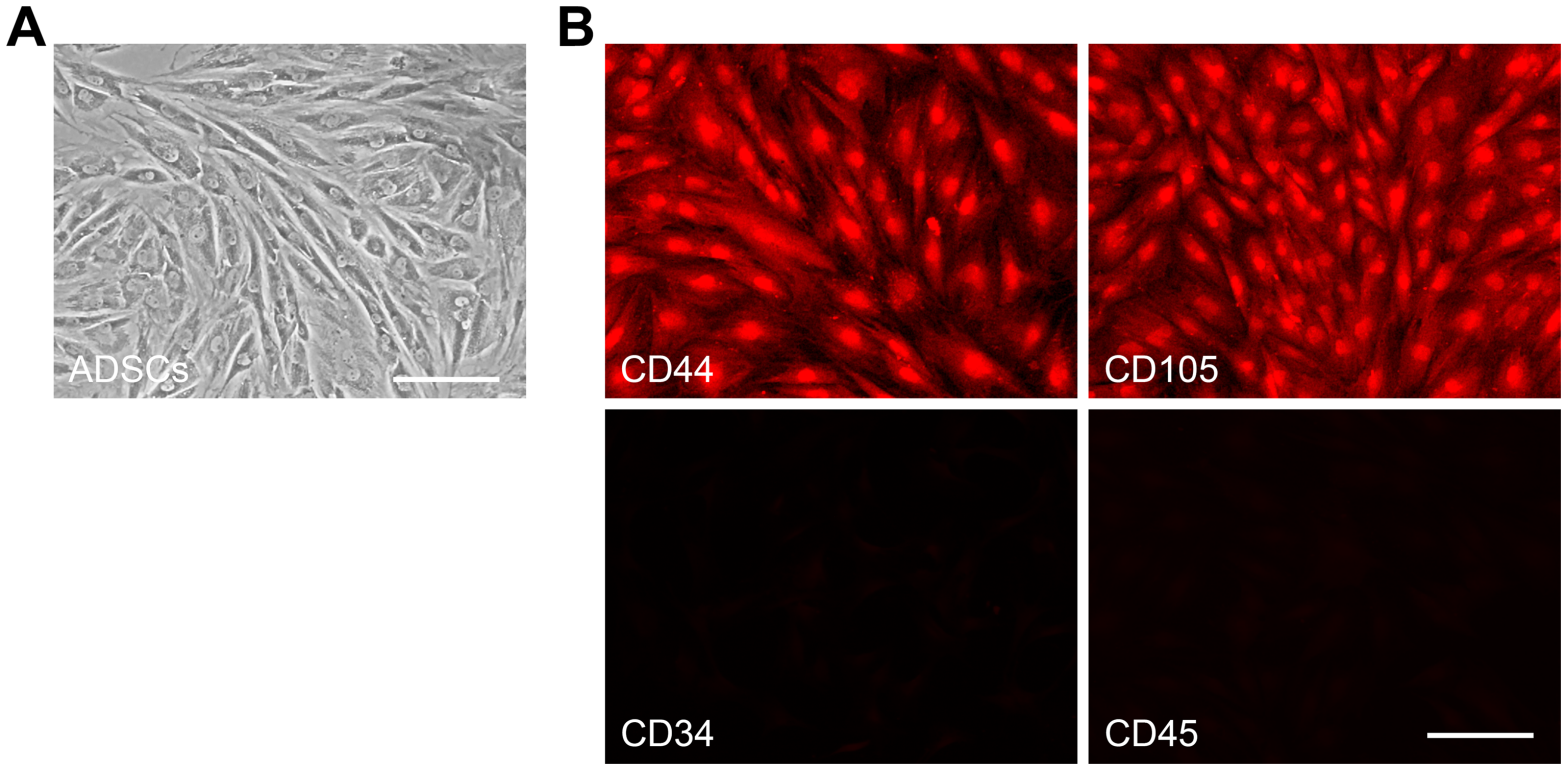
Characteristics of ADSCs. **(A)** Passage 3 of cultured ADSCs showed spindle-shaped fibroblast morphology when observed under an inverted microscope. **(B)** Immunofluorescence staining showed that the ADSCs were positive for the surface antigens CD44 and CD105, while negative for CD34 and CD45. Scale bars: 100 μm **(A,B)**.

Three days after plating, the ADSCs adhered to the bottom of the culture flask and formed scattered colonies. By day 14 of culture, they reached about 90% confluence and exhibited elongated, spindle-shaped morphology. Passaged cells were evenly distributed and achieved full confluence every 3–4 days. After three passages in primary culture, ADSCs formed a monolayer of large, flattened cells. At confluence, cells displayed homogeneous fibroblast-like morphology with prominent spindle features (Figure 1A). Immunophenotypic analysis confirmed that ADSCs were positive for CD44 and CD105, but negative for CD34 and CD45 (Figure 1B), consistent with the standard immunophenotypic profiles of ADSC (Rohden et al., 2021; Gómez-de Frutos et al., 2019).

### 3.2 Activation of JAK1/STAT3 signaling pathway by ADSC-CM *in vivo*

**Figure 2:**
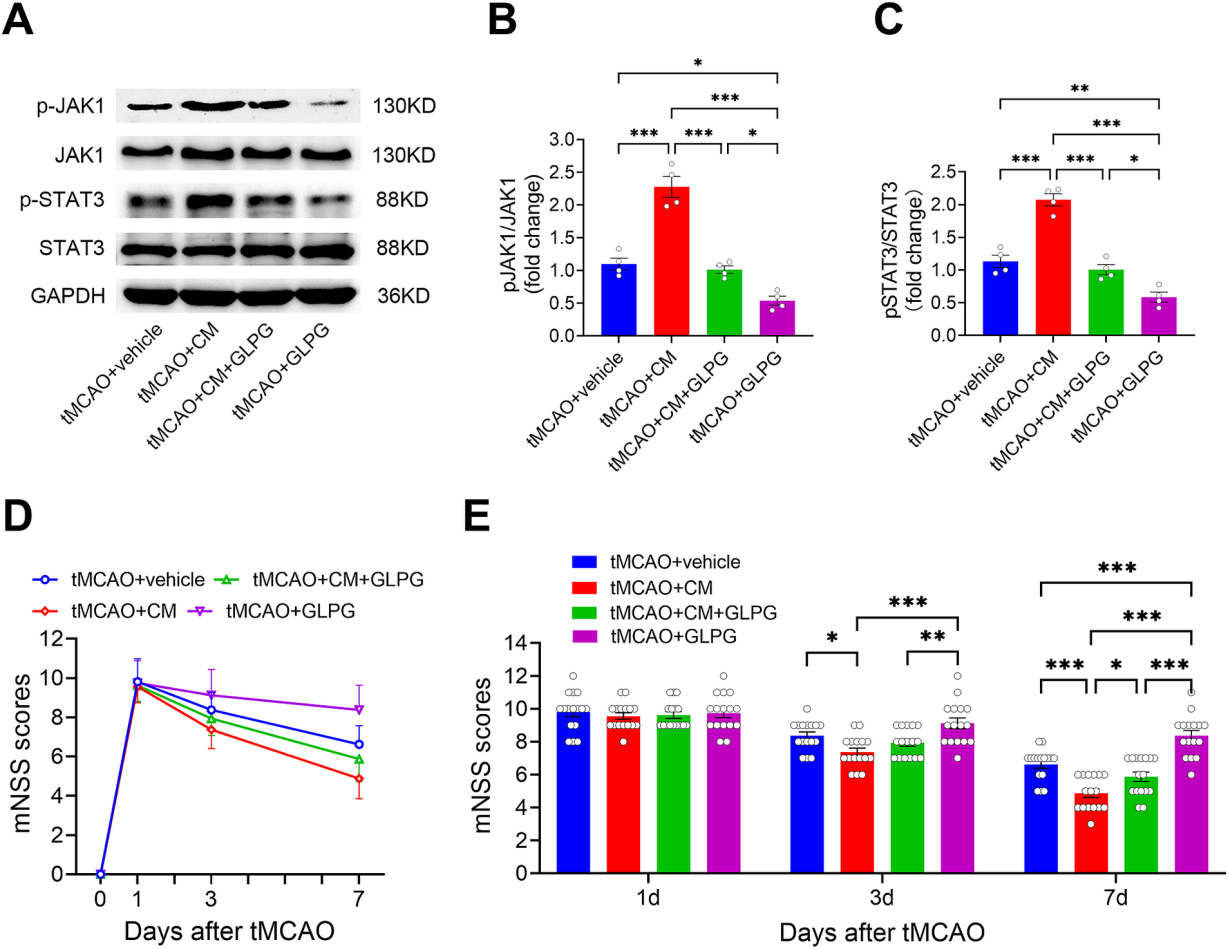
ADSC-CM promotes neurological functional recovery in tMCAO rats through the JAK1-STAT3 signaling pathway. **(A)** Western blot bands of JAK1, pJAK1, STAT3, pSTAT3, and GAPDH for each group. **(B)** Comparison of the relative ratio of pJAK1 to JAK1 across experimental groups (*n* = 4). **(C)** Comparison of the relative ratio of pSTAT3 to STAT3 across experimental groups (*n* = 4). **(D)** The line graph displays the trend in mNSS scores over time (days 1, 3, and 7) after tMCAO in each group. **(E)** The bar graph compares mNSS scores on days 1, 3, and 7 after tMCAO across experimental groups (*n* = 16). Data are expressed as means ± SEM. The differences revealed by western blot and in mNSS scores were assessed by one-way and two-way ANOVA, respectively, both followed by Bonferroni *post hoc* tests. *p < 0.05, **p < 0.01, ***p < 0.001.

To determine whether ADSC-CM activates the JAK1/STAT3 pathway in the ischemic brain, we performed Western blot analysis on protein extracts from the peri-infarct cortex of tMCAO rats. As shown in Figures 2A,B, rats treated with ADSC-CM exhibited a significant increase in the JAK1 phosphorylation, as evidenced by markedly higher pJAK1/JAK1 levels compared to vehicle controls (*p* < 0.001). On the contrary, GLPG0634 mono-treatment significantly attenuated JAK1 phosphorylation (*p* < 0.05). STAT3 phosphorylation followed a similar pattern, with ADSC-CM-treated rats showing notably higher pSTAT3/STAT3 levels than the vehicle-treated rats (*p* < 0.001), while GLPG0634 mono-treatment reduced these levels (*p* < 0.01) (Figures 2A,C). Critically, co-treatment (ADSC-CM + GLPG0634) abolished the ADSC-CM-induced activation, resulting in pJAK1/JAK1 and pSTAT3/STAT3 levels that were not significantly different from the vehicle controls (*p* > 0.05 for both) (Figures 2B,C). These *in vivo* findings demonstrate that ADSC-CM robustly activates the JAK1/STAT3 signaling pathway in the brain following ischemic injury. GLPG0634-mediated inhibition confirms the pathway’s central role in mediating ADSC-CM effects, highlighting its potential therapeutic role in neuroprotection against ischemic injury.

### 3.3 ADSC-CM enhances neurological functional recovery of rats after tMCAO

Longitudinal mNSS assessment demonstrated time-dependent, treatment-specific patterns of functional recovery (Figures 2D,E). In tMCAO rats, vehicle treatment exhibited progressive but limited recovery, whereas ADSC-CM treatment significantly accelerated neurological restoration, reducing deficits by 14% at Day 3 (*p* < 0.05) and by 36% at Day 7 (*p* < 0.001). Co-treatment eliminated these benefits, resulting in mNSS scores that were indistinguishable from those of vehicle controls at both Day 3 and Day 7 (*p* > 0.05 for both). Notably, compared to vehicle controls, GLPG0634 mono-treatment exacerbated deficits by 26% at Day 7 (*p* < 0.001). These findings demonstrate that ADSC-CM can significantly improve neurological function in tMCAO rats, and that GLPG0634 inhibits this effect, confirming the importance of the JAK1/STAT3 signaling pathway in ADSC-CM’s neuroprotective effects.

### 3.4 ADSC-CM reduces infarct volume and brain edema and enhances neuronal survival

**Figure 3:**
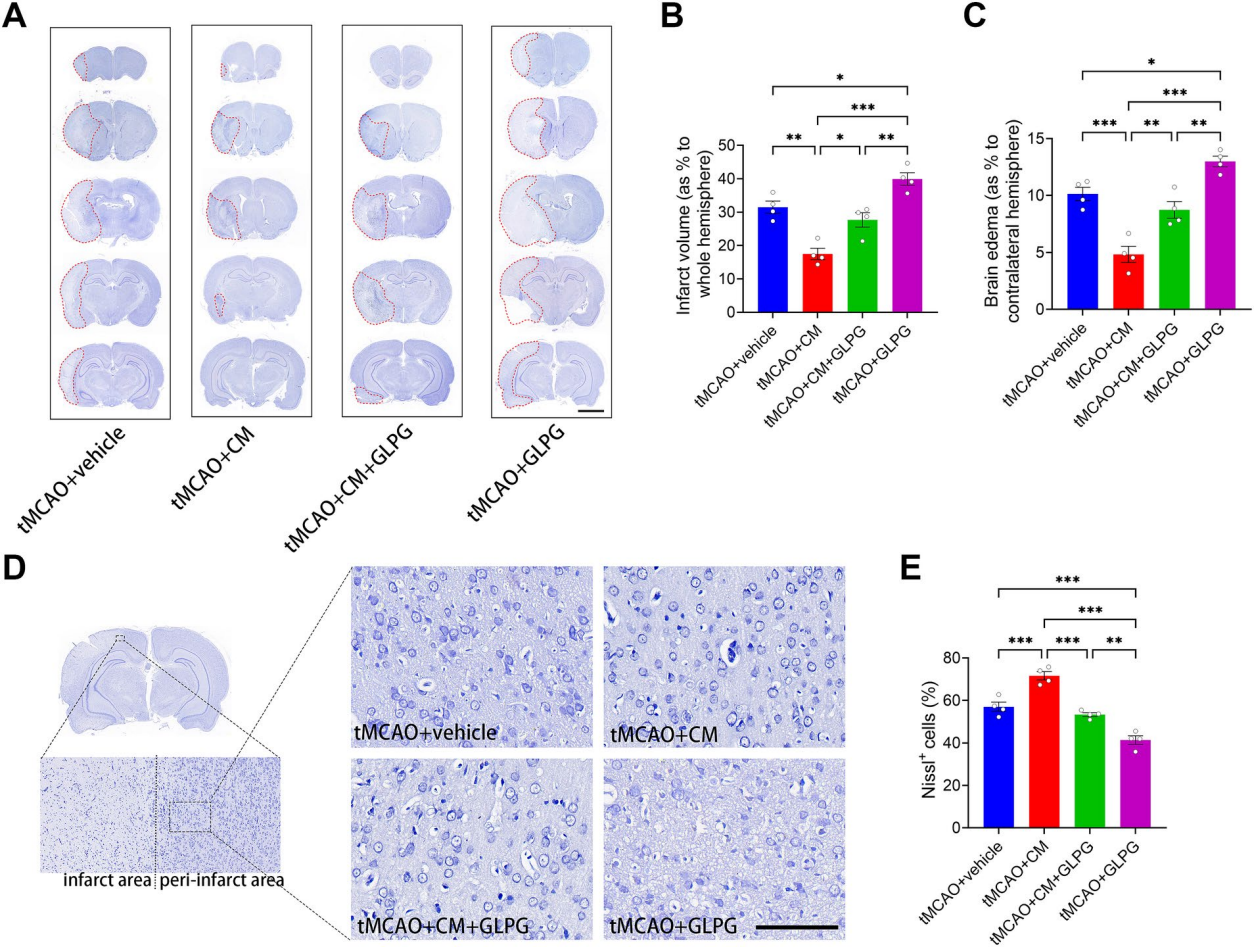
ADSC-CM reduces infarct volume and brain edema and enhances neuronal survival in tMCAO rats through the JAK1-STAT3 signaling pathway. **(A)** Representative coronal sections of the whole brain stained with Nissl from each group. From top to bottom, the slice levels are located at the following distances from the bregma: +3.20 mm, +1.20 mm, −0.80 mm, −2.80 mm, and −4.80 mm. The red dashed lines outline the infarct regions. **(B)** Comparison of the infarct volume as a percentage of the whole hemisphere across experimental groups (*n* = 4). **(C)** Comparison of the brain edema as a percentage of the contralateral hemisphere across experimental groups (*n* = 4). **(D)** Nissl staining of the peri-infarct cortex. Left panel: schematic representation of the region analyzed. Right panel: representative images of Nissl-stained neurons in the peri-infarct cortex of each group. **(E)** Comparison of the percentage of Nissl^+^ cells (indicative of neuronal survival) in the peri-infarct cortex across experimental groups (*n* = 4). Data are expressed as means ± SEM. The difference between the groups was assessed using a one-way ANOVA followed by Bonferroni *post hoc* tests. *p < 0.05, **p < 0.01, ***p < 0.001. Scale bars: 5 mm (A), 50 μm **(D)**.

To evaluate the protective effects of ADSC-CM on ischemic brain tissue, we assessed infarct volume, brain edema, and neuronal survival in tMCAO rats. Nissl staining revealed that treatment with ADSC-CM significantly reduced the cerebral infarct volume by 44% compared to the vehicle controls (*p* < 0.01) (Figures 3A,B). Conversely, GLPG0634 mono-treatment aggravated ischemic damage, increasing the infarct volume by 27% relative to the vehicle controls (*p* < 0.05). The co-treatment group showed no significant difference in infarct volume compared to the vehicle controls (*p* > 0.05), indicating that the protective effect of ADSC-CM was abolished by JAK1 inhibition. Consistent with the reduction in infarct volume, ADSC-CM treatment also significantly ameliorated brain edema compared to the vehicle controls (*p* < 0.001) (Figure 3C). In contrast, GLPG0634 mono-treatment exacerbated brain edema (*p* < 0.05), while the co-treatment group again showed no significant benefit compared to the vehicle group (*p* > 0.05). Analysis of neuronal survival in the peri-infarct cortex exhibited a parallel trend (Figures 3D,E). ADSC-CM-treated rats retained a significantly higher percentage of Nissl^+^ neurons (71.61 ± 3.95%) compared to vehicle controls (57.05 ± 4.37%) (*p* < 0.001). GLPG0634 mono-treatment, however, reduced neuronal survival to 41.35 ± 4.02% (*p* < 0.001 vs. vehicle). The co-treatment group failed to show a significant improvement in neuronal survival over the vehicle controls (*p* > 0.05). Collectively, these findings demonstrate that ADSC-CM confers robust protection against ischemic damage by simultaneously reducing infarct volume, mitigating brain edema, and promoting neuronal survival, all of which are critically dependent on the JAK1/STAT3 signaling pathway.

### 3.5 ADSC-CM promotes nerve fiber regeneration and synaptic plasticity

**Figure 4:**
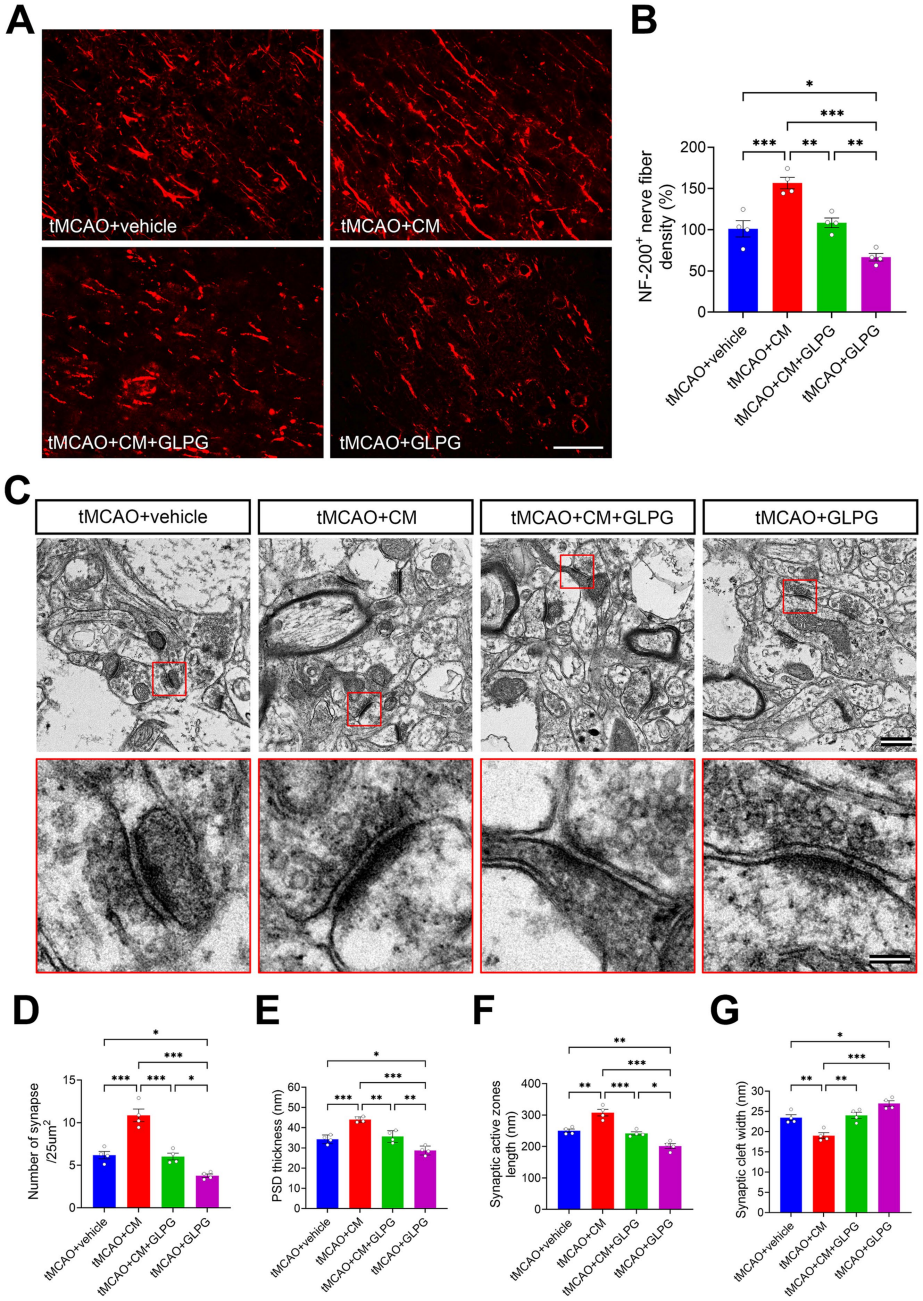
ADSC-CM promotes nerve fiber regeneration and synaptic plasticity in tMCAO rats through the JAK1/STAT3 signaling pathway. **(A)** Representative immunofluorescence images of NF-200 (red) in the peri-infarct cortex of each group. **(B)** Comparison of the NF-200^+^ nerve fiber density across experimental groups (*n* = 4). (**C**) Representative electron micrographs of synaptic ultrastructure in the peri-infarct cortex of each group. The lower panels show magnified views of the areas outlined by red boxes in the respective upper panels. (**D–G**) Comparison of the number of synapses, thickness of PSDs, length of synaptic active zones, and width of synaptic clefts in the peri-infarct cortex across experimental groups (*n* = 4). Data are expressed as the means ± SEM. The difference between the groups was assessed using a one-way ANOVA followed by Bonferroni post hoc tests. *p < 0.05, **p < 0.01, ***p < 0.001. Scale bars: 100 μm **(A)**, 500 nm (**C**, upper panel), 100 nm (**C**, lower panel).

NF-200 is predominantly expressed in nerve fibers, including axons and dendrites, and participates in axonal growth and synaptic function regulation (Ueno et al., 2012). Quantitative analysis of NF-200^+^ nerve fiber density in the peri-infarct cortex at day 7 post-tMCAO revealed profound intervention-specific effects (Figures 4A,B). Compared with vehicle controls, ADSC-CM treatment increased NF-200^+^ nerve fiber density by 55% (*p* < 0.001), demonstrating robust nerve fiber regeneration. However, co-treatment eliminated this benefit, resulting in levels comparable to those of vehicle controls (*p* > 0.05). Furthermore, GLPG0634 mono-treatment increased nerve fiber degeneration, reducing NF-200^+^ nerve fiber density to 66% of control levels (*p* < 0.05), highlighting the harmful effects of JAK1/STAT3 inhibition. TEM analysis further identified JAK1/STAT3-mediated synaptic remodeling in the peri-infarct cortex (Figures 4C–G). Compared with vehicle controls, ADSC-CM treatment significantly ameliorated synaptic ultrastructure, as indicated by elevated synaptic density, thicker PSDs, longer active zones, and narrower synaptic clefts (*p* < 0.001 for all). Conversely, GLPG0634 mono-treatment aggravated synaptic injury, as evidenced by decreased synaptic density (*p* < 0.05), thinner PSDs (*p* < 0.05), shorter active zones (*p* < 0.01), and wider synaptic clefts (*p* < 0.05). Notably, co-treatment did not significantly restore synaptic ultrastructure, with all parameters remaining indistinguishable from vehicle controls (*p* > 0.05). Overall, these results suggest that ADSCs enhance nerve fiber regeneration and synaptic plasticity in tMCAO rats via the JAK1/STAT3 signaling pathway, and GLPG0634 inhibits this effect.

### 3.6 ADSC-CM promotes angiogenesis and improves mitochondrial structure and function

**Figure 5:**
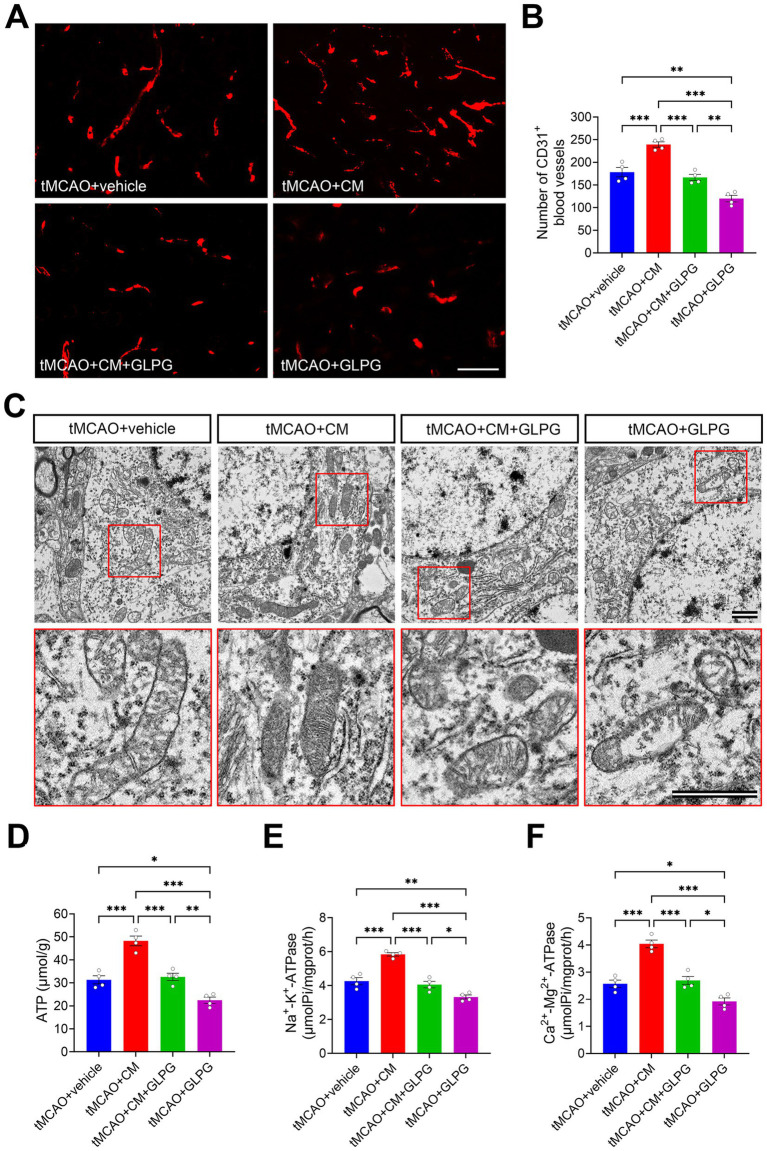
ADSC-CM promotes angiogenesis and improves mitochondrial structure and function in tMCAO rats through the JAK1/STAT3 signaling pathway. **(A)** Representative immunofluorescence images of CD31 (red) in the peri-infarct cortex of each group. **(B)** Comparison of the CD31^+^ vessel density in the peri-infarct cortex across experimental groups (*n* = 4). **(C)** Representative electron micrographs of mitochondrial ultrastructure in the peri-infarct cortex of each group. The lower panels show magnified views of the areas outlined by red boxes in the respective upper panels. **(D–F)** Comparison of the ATP levels, Na^+^-K^+^-ATPase activity, and Ca^2+^-Mg^2+^-ATPase activity in the peri-infarct cortex across experimental groups (*n* = 4). Data are expressed as means ± SEM. The difference between the groups was assessed using a one-way ANOVA followed by Bonferroni post hoc tests. *p < 0.05, **p < 0.01, ***p < 0.001. Scale bars: 100 m **(A)**, 1 μm **(C)**.

CD31, a well-established endothelial cell marker, plays a pivotal role in assessing cerebrovascular pathophysiology. Previous animal model data indicate an increased density of CD31^+^ blood vessels in the peri-infarct region following stroke (Chen et al., 2019; Li et al., 2024). In this study, quantification of CD31^+^ blood vessels by immunofluorescence in the peri-infarct cortex at day 7 demonstrated that the JAK1/STAT3 pathway promoted angiogenesis (Figures 5A,B). ADSC-CM treatment significantly increased blood vessel density compared to vehicle controls (*p* < 0.001). Conversely, GLPG0634 mono-treatment substantially reduced blood vessel density compared with vehicle controls (*p* < 0.01). Co-treatment abolished the pro-angiogenic effect of ADSC-CM, as evidenced by blood vessel density comparable to vehicle controls (*p* > 0.05). Given the critical interplay between mitochondrial integrity and blood supply, the impact of ADSC-CM on mitochondrial structure and function within the peri-infarct cortex was further investigated. As shown in Figure 5C, TEM analysis revealed substantial mitochondrial damage in vehicle controls, characterized by swelling, disrupted membranes, sparse and swollen cristae, and, in some cases, complete loss of cristae. Treatment with ADSC-CM markedly alleviated this damage; however, the protective benefits were abolished by co-treatment. Moreover, tMCAO rats treated only with GLPG0634 exhibited more severe mitochondrial structural damage than vehicle controls. Mitochondrial function was assessed by measuring key parameters related to energy metabolism (Figures 5D–F). Bioenergetic assays demonstrated that ADSC-CM treatment significantly restored ATP levels and increased Na^+^-K^+^-ATPase and Ca^2+^-Mg^2+^-ATPase activities compared to vehicle controls (*p* < 0.001 for all). Notably, co-treatment abolished these beneficial effects, with ATP levels and both ATPase activities showing no significant differences from vehicle controls (*p* > 0.05 for all). Meanwhile, GLPG0634 mono-treatment had an inhibitory effect, resulting in a substantial reduction in ATP production (*p* < 0.05) and both ATPase activities (*p* < 0.01 and *p* < 0.05, respectively) relative to vehicle controls. Collectively, these findings suggest that ADSC-CM promotes angiogenesis and improves mitochondrial structure and function in tMCAO rats by activating the JAK1/STAT3 signaling pathway, an effect inhibited by GLPG0634.

### 3.7 Characteristics of primary cortical neurons and OGD injury

**Figure 6:**
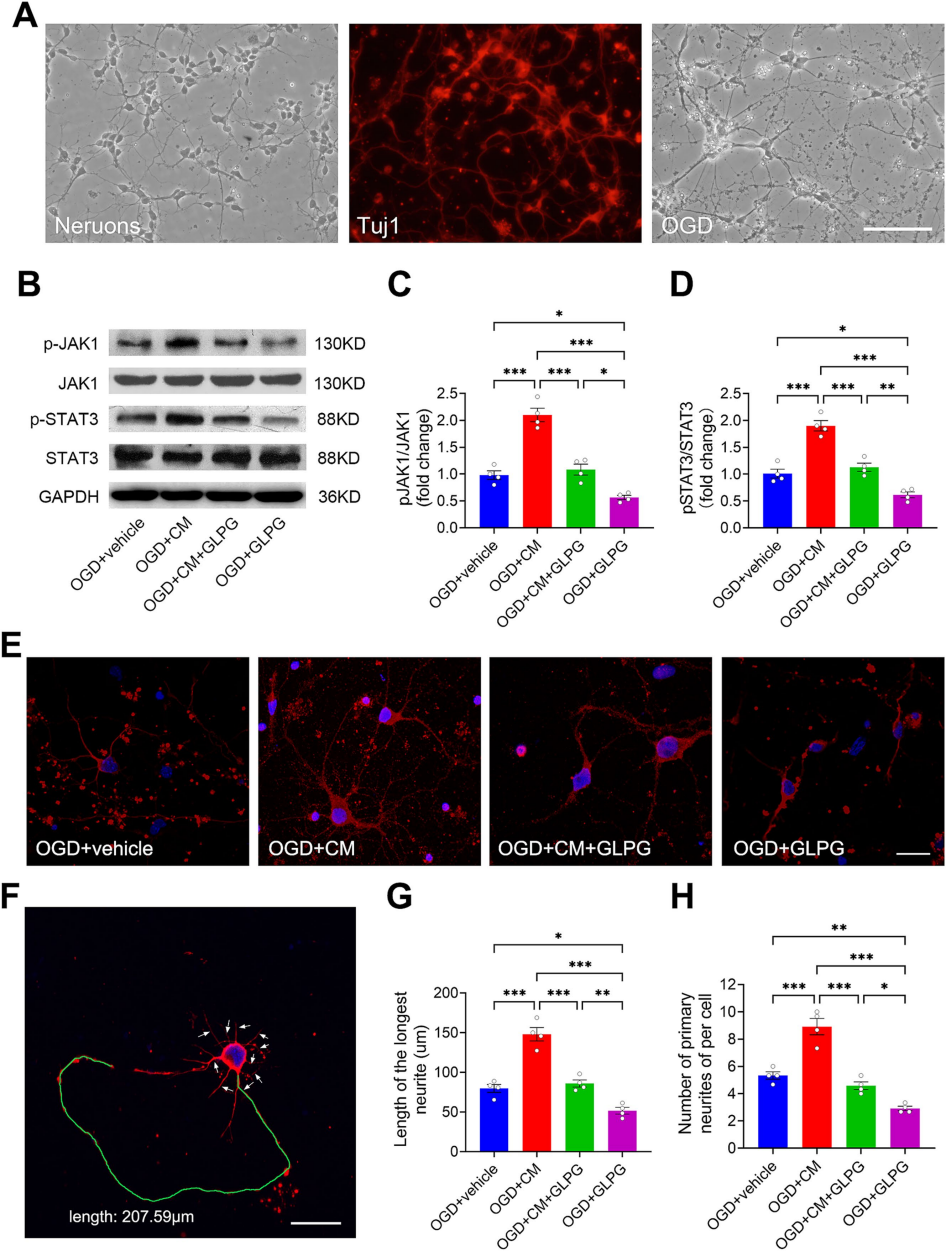
ADSC-CM promotes neurite outgrowth in OGD-injured neurons through the JAK1-STAT3 signaling pathway. **(A)** On day 5 of cultivation, neuronal neurites extended and formed an extensive neural network. Immunofluorescence staining showed that the neurons were positive for the neuronal-specific marker Tuj1. After OGD injury, neuronal neurites were damaged, partially interrupted, and disappeared. **(B)** Western blot bands of JAK1, pJAK1, STAT3, pSTAT3, and GAPDH for each group. **(C)** Comparison of the relative ratio of pJAK1 to JAK1 across experimental groups (*n* = 4). **(D)** Comparison of the relative ratio of pSTAT3 to STAT3 across experimental groups (*n* = 4). **(E)** Immunofluorescence images of neurons from each group, showing the effects of ADSC-CM and GLPG0634 on neurite outgrowth. Neurons were stained with the Tuj1 antibody (red) to label the neuronal cytoskeleton, and nuclei were counterstained with DAPI (blue). **(F)** Diagram illustrating how to measure the length of the longest neurite and the number of primary neurites in neurons. The green line shows the trajectory of the longest neurite, and white arrows point to primary neurites. **(G,H)** Comparison of the length of the longest neurite and the number of primary neurites in neurons across experimental groups (*n* = 4). Data are expressed as means ± SEM. The difference between the groups was assessed using a one-way ANOVA followed by Bonferroni post hoc tests. *p < 0.05, **p < 0.01, ***p < 0.001. Scale bars: 100 μm **(A)**, 20 μm **(E,F)**.

To further investigate the effect of ADSC-CM on neuronal regeneration in a simplified *in vitro* system, we employed primary cortical neurons following OGD injury. After isolation and plating, primary rat cortical neurons initially appeared round and small with minimal neurite outgrowth. They gradually adhered to the culture substrate and displayed a more defined, polarized morphology with minor neurites by 24 h post-plating. By the fifth day *in vitro*, substantial neurite outgrowth was observed, forming an extensive interconnected network across the culture surface (Figure 6A, left panel). Immunofluorescence staining revealed that most cells were positive for Tuj1, indicating high neuronal culture enrichment (Figure 6A, middle panel). Following OGD injury, neurons exhibited marked morphological changes, including neurite retraction, fragmentation, and a notable decrease in network complexity and density compared to normal cultures (Figure 6A, right panel). These observations suggest that OGD induced significant neurite damage, indicating disruption of neuronal connectivity and function.

### 3.8 Activation of JAK1/STAT3 signaling pathway by ADSC-CM *in vitro*

Western blot analysis of OGD-injured neurons demonstrated that ADSC-CM treatment significantly activated the JAK1/STAT3 pathway. Specifically, the pJAK1/JAK1 levels were markedly higher in the ADSC-CM-treated neurons compared to the vehicle controls (*p* < 0.001). In contrast, GLPG0634 mono-treatment significantly suppressed JAK1 phosphorylation in neurons (*p* < 0.05) (Figures 6B,C). STAT3 activation mirrored this trend, with the pSTAT3/STAT3 levels being significantly higher in the ADSC-CM-treated neurons but lower in the GLPG0634-treated neurons than those in the vehicle controls (*p* < 0.001 and *p* < 0.05, respectively) (Figures 6B,D). Importantly, no significant differences were observed between the co-treatment group and the vehicle controls for either pJAK1/JAK1 or pSTAT3/STAT3 levels (*p* > 0.05 for both) (Figures 6C,D). Consistent with *in vivo* findings, these *in vitro* results consistently show that ADSC-CM directly activates the JAK1/STAT3 pathway in ischemic neurons, and this activation is effectively inhibited by GLPG0634.

### 3.9 ADSC-CM promotes neurite outgrowth *in vitro*

Immunofluorescence analysis revealed distinct neuritogenic responses among different treatment groups in OGD-injured neurons (Figure 6E). Compared to vehicle controls, ADSC-CM treatment resulted in a 1.86-fold increase in the length of the longest neurites (*p* < 0.001) (Figure 6G). This enhancement was accompanied by a 1.67-fold increase in the number of primary neurites (*p* < 0.001) (Figure 6H), indicating robust neurite outgrowth. In contrast, GLPG0634 mono-treatment reduced neurite growth to approximately 60% of control levels (*p* < 0.05). Co-treatment showed no significant difference from vehicle controls (*p* > 0.05) (Figures 6G,H). These findings demonstrate that ADSC-CM promotes neurite outgrowth in OGD-injured neurons through the JAK1/STAT3 signaling pathway.

## 4 Discussion

### 4.1 ADSC-CM confers comprehensive neuroprotection via the JAK1/STAT3 signaling pathway

The therapeutic paradigm for stem cell therapy in ischemic stroke has progressively shifted from cell replacement to appreciating the potent paracrine effects of secreted factors (Davis et al., 2021; Su et al., 2025; Song et al., 2024). ADSCs are a particularly attractive source due to their accessibility, abundance, and robust secretome (Zhou J. et al., 2024; Zhang Y. C. et al., 2022; Tang et al., 2024). Aligning with this paradigm, our findings demonstrate that administration of ADSC-CM alone, without the cells themselves, is sufficient to confer comprehensive neuroprotection against ischemic injury in both *in vitro* and *in vivo* models. The observed benefits, encompassing enhanced neurite outgrowth, reduced infarct volume and brain edema, improved neuronal survival, promoted neurovascular remodeling, and preserved mitochondrial function, underscore the multifaceted nature of this protection. Given that ischemic brain injury is inherently complex, such a comprehensive strategy is essential, as it necessitates targeting multiple pathways within the injury cascade (Xia et al., 2025; Lin et al., 2023; Bai et al., 2025). Moreover, we mechanistically delineate that these benefits fundamentally depend on activation of the JAK1/STAT3 signaling pathway. This conclusion is robustly supported by the consistent abolition of the protective effects upon co-treatment with the selective JAK1 inhibitor GLPG0634.

### 4.2 Neuronal survival and circuit reconstruction

Neuronal loss is a major contributor to the functional deficits observed after ischemic stroke (Gong et al., 2022; Zhao et al., 2022). For this reason, the primary goals of thrombolysis and mechanical thrombectomy are to restore cerebral blood flow and reperfuse the infarcted area, thereby preventing the expansion of infarct volume and reducing neuronal death (Fang et al., 2024; Zhao et al., 2022). In the present study, we found that ADSC-CM treatment significantly reduced infarct volume and improved neuronal survival in the peri-infarct cortex, consistent with the known therapeutic effects of MSC-based therapies (Wang et al., 2023; Zhang L. et al., 2022). These effects likely arise from a synergistic combination of anti-apoptotic signals, attenuation of inflammatory responses, and direct activation of pro-survival pathways within stressed neurons (Zhu P. et al., 2024; Zhang Y. C. et al., 2022). However, functional recovery after ischemic stroke extends beyond preserving the neurons; it equally depends on rebuilding the intricate network of connections essential for neural circuit function (Wu et al., 2024; Joy and Carmichael, 2021). Our data significantly advance the field by demonstrating that ADSC-CM not only supports neuronal survival but also actively promotes the restoration of neuronal connectivity. This is evidenced *in vivo* by the significant increase in NF-200^+^ nerve fiber density, and *in vitro* by the rescue of oxygen–glucose deprivation (OGD)-induced neurite retraction. Furthermore, through detailed ultrastructural analysis using TEM, we provide direct and unambiguous evidence that ADSC-CM treatment preserves and enhances synaptic integrity. The increased synaptic density, thicker PSDs, longer active zones, and narrower synaptic clefts observed in the ADSC-CM group are hallmarks of enhanced synaptic plasticity. The strong correlation between these structural improvements and accelerated neurological functional recovery (as measured by the mNSS) suggests a direct causal link, by which JAK1/STAT3-mediated neuronal survival and synaptic repair contribute to the amelioration of functional deficits.

### 4.3 Coordination of neurovascular repair, edema reduction, and mitochondrial recovery

A particularly insightful finding of our study is the coordinated improvement in angiogenesis, reduction of brain edema, and recovery of mitochondrial function, which collectively outline a virtuous cycle of repair orchestrated by ADSC-CM via the JAK1/STAT3 pathway. The significant increase in CD31^+^ vessel density in the peri-infarct region underscores the pro-angiogenic capacity of ADSC-CM. This process is vital for re-establishing a functional microvasculature, thereby restoring the delivery of oxygen and nutrients to the metabolically compromised penumbra (Fang et al., 2023; Wang et al., 2024). Meanwhile, the pronounced alleviation of brain edema suggests a stabilization of the BBB, reducing vasogenic edema, and a mitigation of energy failure, alleviating cytotoxic edema—the latter being directly supported by the restored Na^+^-K^+^-ATPase activity (Zhu J. et al., 2024; Hellas and Andrew, 2021). We posit that the promotion of angiogenesis contributes to restoring a competent vascular network, which in turn reduces vascular leakage and edema formation (Ghori et al., 2022). By restoring vascular integrity and mitigating brain edema, ADSC-CM establishes a more favorable tissue microenvironment that provides the foundation for the recovery of cellular metabolic machinery. Our data provide direct evidence for this, showing that ADSC-CM treatment robustly ameliorated ischemic mitochondrial damage, as evidenced by restored ultrastructure, increased ATP production, and enhanced Na^+^-K^+^-ATPase and Ca^2+^-Mg^2+^-ATPase activities. The JAK1/STAT3 dependence of all three processes (angiogenesis, edema reduction, and mitochondrial recovery) indicates a unified mechanistic underpinning. Thus, a compelling narrative emerges: ADSC-CM, via JAK1/STAT3 activation, promotes angiogenesis and BBB stabilization, which reduces edema and establishes a conducive microenvironment. This improved milieu supports the repair of mitochondria, the cellular power plants. The subsequent restoration of bioenergetic capacity then furnishes the ATP necessary to fuel the energy-demanding processes of axonal regeneration, synaptic plasticity, and the maintenance of the newly formed vasculature itself. This positive feedback loop ultimately underpins the significant neurological recovery observed in ADSC-CM-treated rats.

### 4.4 Context-dependent role of JAK1/STAT3 signaling

The role of the JAK/STAT3 pathway in cerebral ischemia is complex and context-dependent, with the literature reporting a paradoxical duality of both neuroprotective and detrimental outcomes (Xi et al., 2025; Kumari et al., 2024; Zhang et al., 2024). Our results, utilizing the selective JAK1 inhibitor GLPG0634, clearly demonstrate a protective role in the specific context of ADSC-CM application. The complete abolition of ADSC-CM benefits across all assessed parameters, ranging from histology to behavior, firmly establishes that activation of this pathway is a necessary condition for the observed neuroprotection. The discrepancy between our findings and studies reporting detrimental effects of STAT3 activation may be explained by the nature of the upstream activators. ADSC-CM represents a complex, physiological cocktail of numerous factors (e.g., growth factors, cytokines) (Ya et al., 2024; Tian et al., 2024), which may activate the JAK1/STAT3 pathway in a spatiotemporally coordinated and balanced manner. This “balanced activation” by a physiological mix of factors, potentially including IL-10, BDNF, and VEGF, may steer the pathway toward pro-survival and reparative genomic responses. This is in contrast to the sustained, potent, yet likely unregulated activation that might occur in pure inflammatory responses or under different pathological conditions (Xu et al., 2023; Kumari et al., 2024). It is, however, imperative to interpret our pharmacological data with precision. While GLPG0634 abolished the benefits of ADSC-CM, establishing JAK1/STAT3 as a necessary pathway, our data do not preclude the potential concurrent activation of other parallel signaling pathways (e.g., phosphatidylinositol-3-kinase/AK strain transforming [PI3K/Akt], mitogen-activated protein kinase/extracellular signal-regulated kinase [MAPK/ERK]) by other constituents of the CM. The pleiotropic nature of the ADSC secretome suggests a signaling network, with JAK1/STAT3 as a key, non-redundant node.

### 4.5 Limitations and future directions

Despite the promising findings, several limitations must be acknowledged to guide future research. First, the specific bioactive composition of the ADSC-CM used in this study was not characterized. Identifying the key effector molecules within the secretome responsible for JAK1/STAT3 activation is essential for therapeutic development, as it would enable standardization, quality control, and potentially the engineering of a more effective and defined biologic. Future studies employing proteomic analysis, followed by factor depletion or neutralization experiments, are warranted. Second, the use of bulk tissue homogenates for Western blot analysis precludes the identification of the specific cell types (e.g., neurons, astrocytes, endothelial cells) in which the JAK1/STAT3 pathway was activated. The observed effects likely result from cell-specific and potentially interactive signaling. Thus, a critical future direction will be to employ cell-type-specific techniques, such as conditional knockout models and co-localization immunofluorescence, to definitively resolve which cellular subsets are necessary and sufficient for the observed benefits. Third, although Nissl staining is widely used (Liu et al., 2025; Zhou Y. et al., 2024), it provides only morphological criteria and lacks molecular specificity for neuronal death pathways. Future investigations incorporating TUNEL and cleaved-caspase-3 analysis will further dissect the anti-apoptotic mechanisms of ADSC-CM. Fourth, while the statistical power was sufficient to detect the primary effects reported, the sample size for histological and molecular analyses was relatively small. Future investigations with larger cohort sizes would be valuable to confirm these findings and provide greater power for detecting more subtle effects. Fifth, the study’s observation period was limited to 7 days post-ischemia. While significant recovery was observed, longer-term studies are necessary to confirm the sustainability of the functional benefits, assess the stability of the structural repairs, and rule out any potential long-term adverse effects. Furthermore, future investigations should explore optimal dosing regimens, therapeutic windows, and the potential synergistic effects of combining ADSC-CM with existing reperfusion therapies, such as rt-PA.

## 5 Conclusion

In summary, our integrated multi-level analysis provides compelling evidence that ADSC-CM elicits profound and multifaceted neuroprotection against ischemic stroke by activating the JAK1/STAT3 signaling pathway. This activation initiates a coordinated repair program that involves neuronal salvage, neurovascular regeneration, synaptic plasticity, and metabolic recovery. The consistent abrogation of these benefits by GLPG0634 firmly positions this pathway as an integral mechanistic hub. These findings not only elucidate a key molecular mechanism underlying ADSC-based therapy but also open new avenues for therapeutic intervention. They suggest that strategies aimed at either enhancing the endogenous reparative functions of ADSCs or developing small-molecule modulators that safely target the JAK1/STAT3 axis could hold significant promise for improving outcomes after ischemic stroke.

## Data Availability

The original contributions presented in the study are included in the article/supplementary material, further inquiries can be directed to the corresponding author.
